# No influence of oxygen levels on pathogenesis and virus shedding in Salmonid alphavirus (SAV)-challenged Atlantic salmon (*Salmo salar *L.)

**DOI:** 10.1186/1743-422X-7-198

**Published:** 2010-08-21

**Authors:** Linda Andersen, Kjartan Hodneland, Are Nylund

**Affiliations:** 1 Department of Biology, University of Bergen, Pb 7800, N-5020 Bergen, Norway; 2 Cavanilles Institute of Biodiversity and Evolutionary Biology, University of Valencia, Pb 22085, 46071 Valencia, Spain

## Abstract

**Background:**

For more than three decades, diseases caused by salmonid alphaviruses (SAV) have become a major problem of increasing economic importance in the European fish-farming industry. However, experimental infection trials with SAV result in low or no mortality i.e very different from most field outbreaks of pancreas disease (PD). This probably reflects the difficulties in reproducing complex biotic and abiotic field conditions in the laboratory. In this study we looked at the relationship between SAV-infection in salmon and sub-lethal environmental hypoxia as a result of reduced flow-through in tank systems.

**Results:**

The experiment demonstrated that constant reduced oxygen levels (60-65% oxygen saturation: 6.5-7.0 mg/L) did not significantly increase the severity or the progress of pancreas disease (PD). These conclusions are based upon assessments of a semi-quantitative histopathological lesion score system, morbidities/mortalities, and levels of SAV RNA in tissues and water (measured by 1 MDS electropositive virus filters and downstream real-time RT-PCR). Furthermore, we demonstrate that the fish population shed detectable levels of the virus into the surrounding water during viraemia; 4-13 days after i.p. infection, and prior to appearance of severe lesions in heart (21-35 dpi). After this period, viral RNA from SAV could not be detected in water samples although still present in tissues (gills and hearts) at lasting low levels. Lesions could be seen in exocrine pancreas at 7-21 days post infection, but no muscle lesions were seen.

**Conclusions:**

In our study, experimentally induced hypoxia failed to explain the discrepancy between the severities reported from field outbreaks of SAV-disease and experimental infections. Reduction of oxygen levels to constant suboptimal levels had no effect on the severity of lesions caused by SAV-infection or the progress of the disease. Furthermore, we present a modified VIRADEL method which can be used to detect virus in water and to supplement experimental infection trials with information related to viral shedding. By using this method, we were able to demonstrate for the first time that shedding of SAV from the fish population into the surrounding water coincides with viraemia.

## Background

Diseases caused by salmonid alphaviruses; SAV (*Alphavirus*, Togaviridae) have become an increasing problem of economical importance to the European fish-farming industry. Salmonid alphavirus (SAV) is the only alphavirus that has been isolated from fish, and are thought to comprise at least six subtypes (SAV1-6) [1]. Whereas all subtypes have been associated with pancreas disease (PD) affecting Atlantic salmon (*Salmo salar *L.) in sea water [1], SAV2 is the only subtype that is known to cause disease outbreaks in fresh water, i.e in rainbow trout *Oncorhynchus mykiss *(Walbaum) [1-6]. In Norway, SAV3 is the only identified subtype [6-8], and the virus has been shown to affect sea water reared rainbow trout and salmon [9,10].

During PD-outbreaks, affected fish will often exhibit abnormal swimming behaviour and may congregate in net pen corners close to the surface [7]. Affected fish may seem lethargic with a marked loss in appetite. Few if any distinctive gross pathological changes can be seen during experimental SAV-infections. Histopathological findings associated with infections by all subtypes of SAV are very similar [11,12], and may include severe degeneration of exocrine pancreas together with myopathy of heart- and skeletal muscle, with variable inflammation. These significant lesions occur in a sequential manner, with pancreas being the first tissue showing pathology, followed by lesions in heart and skeletal muscle [13,14].

Mortality rates associated with SAV-infections in sea water reared salmon and rainbow trout are highly variable [10,15,16] and range from subclinical infections with no outbreaks [17] to acute outbreaks with high mortality [1,10,18]. The severity of PD in sea water can be affected by a range of factors linked to the environment, pathogen and/or the host such as stressors related to handling, management strategies, other infectious agents [19-21] temperature [18] and differences in genetic factors related to the host or the virus (virulence traits) [15,22-24]. In experimental trials with SAV, however, high mortalities are rarely seen [4,18,22,25-28] probably due to problems with reproducing complex field conditions in the laboratory.

Our understanding on how variations in water temperature, oxygen and salinity levels might influence fish welfare and susceptibility for infectious diseases is limited [29,30]. In general, hypoxia has a negative impact on important mechanisms such as growth, appetite, disease resistance and welfare of salmon [31]. Shortage of oxygen (hypoxia) can act as a stressor to fish [32] and elicit primary stress responses such as release of catecholamines and corticosteroids (see [33,34]) possibly affecting immune responses which renders the fish more susceptible to infections [32-34]. Fish reared in marine net pens/large cage systems experience periods with environmental hypoxia, especially during rapid growth in combination with high stocking densities and high temperatures [35]. Also, oxygen levels within a fish farm may fluctuate with depth and time and within and between sea-cages and due to shifting changes in environmental factors such as water currents, wind, temperature, salinity, oxygen mixing and oxygen production by photosynthetic algae (see [35]). Experimental trials where Atlantic salmon were repeatedly exposed to graded hypoxia have shown that fluctuations between normoxia and 60-65% oxygen saturation is suboptimal for salmon, whereas fluctuations between normoxia and 50% saturation or less have been shown to affect appetite in a negative manner and lead to an increased number of skin lesions and elevation of stress responses (Mette Remen, IMR, Bergen, Norway, *personal communication*). In our study, we wanted to see if by reducing oxygen levels to constant environmentally sub-lethal levels (60-65%) this would affect the development and/or the severity of SAV-infection/PD. This was assessed by measuring levels of SAV in tissues and in water (shedding of virus) by real-time RT-PCR, and by comparing histopathological lesions (heart, pancreas and somatic muscle) and mortalities between the respective groups.

## Materials and methods

### Fish and experimental design

Fish were supplied by a local fish supplier (Hordaland County) and reared at the fish facility at Industrilaboratoriet (ILAB) located at Bergen High Technology Centre, Norway. Prior to the experiment, gills from 30 fish were screened by real-time RT-PCR for the presence of various disease causing agents (SAV, infectious pancreatic necrosis virus (IPNV), infectious salmon anaemia virus (ISAV), *Chlamydia sp*., *Neoparamoeba sp*., *Paranucleospora theridion*, and *Parvicapsula *sp.) with negative results. The fish had a mean weight of 73.2 grams and a mean length of 18.1 cm (n = 30) at the beginning of the experiment and had been vaccinated with a multivalent vaccine (no SAV component). Initially, the fish were reared in fresh water in a flow-through system. The fish group were then exposed to an increasing salinity level (particle filtered (50 μm) and UV-sterilized (> 60 mW/cm^2 ^) sea water), experiencing full salinity 33‰ (mean 31.97‰, range 30.3-32.8‰) and 12 °C (mean 11.95 °C, range 11.6-12.9°C) five weeks prior to the experiment. Salinity, oxygen levels and temperature were monitored at least daily throughout the experiment, and the fish were hand fed daily with a commercial feed. The flow-through in tanks was from 100-400 Lh^-1^tank^-1 ^dependent on desired oxygen levels for the various experimental groups (60-65% - or 85-90% saturation) and according to biomass and temperature. Two hundred and sixty fish were divided into 4 tanks (0.15 m^3^), n = 65 fish per tank. When the experiment started the fish groups had been acclimatized to laboratory conditions for 55 days. The experiment was approved by the Norwegian Animal Research Authorities (NARA) in 2008 (reference number 899).

### Inocula

Supernatants from Chinook salmon embryo (CHSE-214) cell culture (uninfected or salmonid alphavirus (SAV)-infected) were diluted 1:10 in Eagle's Minimum Essential Medium (EMEM) and sterile filtered. Two control groups (2 tanks, n = 65 per tank) were intraperitoneally (i.p.) injected with 0.2 ml of supernatants from uninfected cells whereas two other groups (2 tanks, n = 65 per tank) were i.p. injected with 0.2 ml of supernatants from SAV-infected cells, prepared as described. The SAV-isolate was a SAV subtype 3 isolate; SAVH30/04 (kindly provided by M. Karlsen, University of Bergen). All fish were anaesthetized with Metacaine MS-222 prior to injection. The SAV3 isolate SAVH30/04, originating from salmon in Hordaland county in 2004, had been passed six times in CHSE-214 cell culture until cytopathogenic effect (CPE) could be observed (10 dpi), and had a viral endpoint titer TCID_50 _of 5.6 × 10^4 ^virus per ml in the inoculum.

### Virus end point-titration by indirect fluorescence antibody test (IFAT)

Virus end point-titration was based upon the method described by Kärber [36] and was used to estimate the 50% tissue culture infectious dose (TCID_50_) of the inoculum. Briefly, a ten fold dilution series of the inoculum in medium with 2% Fetal Bovine Serum (FBS) was prepared and inoculated onto rainbow trout (RT)-gill cells grown in a 96-well plate and incubated for 8 days at 14°C. The IFAT procedure was performed as described by [37] but with primary recombinant polyclonal antibodies E2-pTe200 raised against SAV3 (1:400). A panel of four polyclonal recombinant antibodies were generated in 2005 (Karl F. Ottem & Katrine Bones Enger, University of Bergen, *unpublished*) by immunizing rabbits with SAV3-derived recombinant antigens (E1 and E2-region) expressed in *Escherichia coli*, and one of these was used in this study (E2-pTe200)(courtesy of KF. Ottem, University of Bergen). The cells were incubated with a secondary antibody, Alexa Fluor 488 goat anti-rabbit IgG (1:1000 dilution, Molecular probes) and examined in a Leica DMIRBE inverted fluorescence microscope. Cells that had been inoculated with cell media only acted as negative controls.

### Oxygen levels

Two days after SAV-infection, the oxygen levels in two of the tanks (parallel tanks; one uninfected control group and one SAV-infected) were gradually lowered (during the first 24 h period) from normal oxygen conditions (85-90% oxygen saturation, 9.2-9.7 mg O_2 _per liter, approx. 300-400 Lh^-1^tank^-1^) to constant suboptimal/sub-lethal conditions (60-65% oxygen saturation, 6.5-7.0 mg O_2 _per liter, approx. 100-200 Lh^-1^tank^-1^) by slowly reducing flow-through in the tanks. The oxygen levels were monitored closely in the beginning, and then at least daily throughout the experimental period of 70 days. The flow-through was adjusted according to biomass during the experiment in order to keep the oxygen levels at a constant reduced level of 60-65% saturation.

The experimental groups are hereafter throughout the manuscript referred to as CNorm and CRed for the uninfected controls reared at normal and reduced oxygen levels, whereas the SAV-infected groups held under normal and reduced oxygen will be referred to as SNorm and SRed, respectively.

### Sampling of tissues

At various times post SAV-infection (7, 14, 21, 35, 49 and 70 days post infection), tissues were sampled from five fish from each experimental group (CNorm, CRed, SNorm and SRed). Fish were killed by a blow to the head, and blood was taken from the caudal veins into heparinised tubes. Weight/fork length together with gross pathology were noted for all individuals. Fulton's condition factor (K) was calculated by K = W (weight in grams)/L (length in cm)^-3 ^* 100. The samples were kept on ice or fast frozen in liquid nitrogen for real-time RT-PCR (gills and hearts), and fixed by immersion in a modified Karnovsky's fixative for histology (heart, somatic muscle at the level of the lateral line and the dorsal fin, together with pyloric caeca region and spleen with pancreatic tissue). Blood was centrifuged at 1000 × *g *for 5 min and plasma was removed and frozen at -80°C for subsequent SAV RNA real-time RT-PCR measurements. Gills from dead and moribund fish were also analyzed with real-time RT-PCR. In addition, pancreatic tissue, heart and muscle were also processed for histology from moribund fish.

### Bacteriologial examination

Inocula from head kidney of all individuals were plated onto Difco™ Marine Agar 2216 and blood agar supplemented with 1.5% NaCl. Agar was incubated at 15°C until colonies could be seen, or discarded after 14 days if no colonies appeared. Colonies were cultivated in Difco™ Marine Broth 2216 for 24-48 h and frozen in this media with 20% glycerol at -80°C for long term storage of bacteria stock. DNA was extracted from bacteria by resuspending a single colony in 50 μl of destH_2_0, vortexing, heating at 95°C for 5 min and centrifuging at 12000 × *g *for 1 min. One μl of the resulting supernatant containing DNA was used as a template in a PCR reaction; 5 μl of 10 × ExTaq buffer (TaKaRa), 4 μl of 10 mM dNTP's, 1 μl of forward and reverse primers targeting the 16 S rRNA gene of a broad spectra of bacteria; EUGB27F (5'-AGAGTTTGATCMTGGCTCAG-3') and EUG1518R (5'-AAGGAGGTGATCCANCCRCA -3') [38], 0.3 μl of Ex *Taq *polymerase (TaKaRa), to a total volume of 50 μl. The PCR was run under the following conditions; an initial denaturing at 94°C for 3 min, followed by 35 cycles of denaturation at 94°C for 30 s, annealing 52°C for 45 s, elongation 72°C for 2 min, followed by a final elongation stage of 72°C for 10 min. PCR-products were evaluated on a 1% agarose gel in 1 × Tris-acetate-EDTA (TAE) buffer and products were sequenced directly in both directions by the use of a ABI Prism BigDye™ Terminator Cycle Sequencing Ready Reaction kit, version 3.1 (Applied Biosystems, Perkin-Elmer) according to the manufacturers instructions, with the primers EUGB27F or EUG1518R and analyzed at the sequence facility at Bergen High Technology Centre. The sequences were processed using Vector NTI Contig suite version 9.0.0 (Informax) and identified using BLAST.

### Exogenous control for real-time RT-PCR analysis of plasma and water samples

Two exogenous controls or spikes were used in this study in order to quantify SAV-specific viral RNA levels in water or plasma; the extreme halophile *Halobacterium salinarum *(an archaeon) (type strain DSM 3754/ATCC 33171) and the aquatic rhabdovirus Viral Haemorrhagic Septicaemia Virus (VHSV). The VHSV virus isolate was of genotype III and had been isolated from rainbow trout in Norway in 2008 [39], and given the name FA28.02.08 (Genbank acc.# GU121099 and GU121100). *H. salinarum *was cultivated at 37°C in medium 97 from DSMZ to an optical density OD_600 nm _of 2.0. VHSV was cultivated at 14°C in RT-gill cells for two passages until appearance of CPE and with a viral endpoint titer of 1 × 10^7 ^virus per ml. Virus supernatants (sterile filtered) and *H. salinarum *in its medium were aliquoted at these concentrations and frozen at -80°C for subsequent spiking of samples. Plasma samples (25 μl) were added 4 μl of VHSV prior to RNA extraction. For water samples, the *H. salinarum *spike was added prior to filtration (20 μl per liter) as a filtration control, whereas VHSV was added after filtration as a RNA extraction control (4 μl per 350 μl sample of lysis buffer-see next section).

### Water sampling

One liter of sea water was sampled from the respective fish tanks in sterile autoclaved screw-cap bottles (Nunc). Sampling was done at the following time points after SAV infection; 6, 13, 20, 28, 37 and 69 dpi for all tanks. In addition, in order to obtain more detailed information about the onset and duration of the virus shedding period, water from the CNorm and SNorm groups were monitored more closely than CRed and SRed, with water sampling at 2, 4, 8, 10, 15, 17 and 22 days post SAV-infection.

Water filtration for viruses was done according to a VIRADEL (virus-adsorption-elution) method (see [40,41]) using electropositive 1 MDS filters [42,43]. Filtration was done following the instructions made by the manufacturer, with some modifications. Briefly, one liter of sea water was vacuum filtered through one-layer of electropositive Zeta Plus^® ^Virosorb^® ^1 MDS Filters (Cuno Inc, U.S.A.) with a glass filtration system for 47 mm diameter membranes (Pyrex^®^Laboratory Glassware, U.K) with a water flow of 0.2-0.5 liters per min, after adding 20 μl of *H. salinarum *(see previous section). The filters were placed upside down in 1.4 ml of lysis buffer (E.Z.N.A total RNA kit from OmegaBioTek) in 50 mm diameter petri dishes, sealed with parafilm and shaken for 10 minutes (150 rpm) at room temperature. Two portions (à 350 μl) of lysis buffer were removed and 4 μl of VHSV was added to one of these portions (the other portion acted as a VHSV-negative control). The samples were each mixed with 350 μl 70% EtOH, vortexed and frozen at -80°C prior to subsequent thawing and RNA extraction following the manufacturer's protocol using the E.Z.N.A total RNA kit from OmegaBioTek. This modified method was evaluated prior to use and resulted in at least a 20 fold concentration of viral RNA compared to unfiltered (unconcentrated) samples, and was highly reproducible (data not shown).

### RNA extraction and real-time RT-PCR

Total RNA was extracted from tissues (gills and hearts 10-20 μg) using TRIreagent (Sigma) according to the method described by Devold and coworkers [44], whereas total RNA were extracted from serum samples (25 μl) with the E.Z.N.A total RNA kit from OmegaBioTek following the manufacturer's protocol. The RNA was eluted in 50 μl of DEPC treated H_2_0 water. The Verso™ 1-step QRT-PCR Rox kit from Thermo Scientific was used for real-time RT-PCR analysis. The nsP1-assay targeting the nsP1-gene in SAV [45] was applied for specific detection of SAV, whereas the elongation factor 1 alpha (EF1A_A_) assay [46] were used as an endogenous control for tissues. The VHSV08-assay targeting VHSV [39] and the Sal-assay targeting *H. salinarum *(present study; F-primer: 5'-GGGAAATCTGTCCGCTTAACG-3', R-primer: 5'- CCGGTCCCAAGCTGAACA-3', Probe: VIC-5'- AGGCGTCCAGCGGA-3'-MGB) was used as exogenous controls when extracting RNA from plasma and water samples. The Sal-assay generates a 59 bp PCR-product (position 541-600 of Acc.# AB219965). The real-time master mixture consisted of 6.25 μl 1-step QPCR Rox Mix (2 ×), 0.625 µl RT Enhancer, together with 0.125 µl of Verso Enzyme mix. Primer and probe concentrations had been optimized for each assay; F primer/R primer/probe: nsP1 assay (SAV): 900 nm/900 nm/200 nm, EF1A_A _assay (elongation factor 1 alpha); 900 nm/900 nm/225 nm), Sal assay: 300 nm/900 nm/200 nm, VHSV08 assay: 600 nm/600 nm/225 nm. Primers and probes at their respective concentrations were added to the master mixture and adjusted with ddH_2_0 to a total volume of 10.5 µl prior to adding 2 µl of RNA template. The real-time RT-PCR reaction was run in a 7500 Fast Real-Time PCR System cycler from Applied Biosystems using the following conditions: reverse transcription at 50°C for 15 minutes followed by activation of the Thermo-Start DNA polymerase at 95°C for 15 minutes prior to amplification with 45 cycles of 95°C for 15 seconds and 60°C for 1 min (denaturation and annealing/extension). For each assay a standard curve was generated from dilution series of RNA in 20 ng/μl yeast tRNA (Invitrogen) (nsP1-, EF1A_A_- and Sal-assays) or ddH_2_0 (VHSV-assay).

All samples that were analyzed with real-time RT-PCR were performed in triplicate. Only samples that were positive in triplicates were considered for normalization. Thresholds for all assays were set to 0.01 except for the Sal-assay which was set to 0.001. Ct-values obtained for the target gene (nsP1-assay) were normalized against the endogenous control EF1A_A_-assay (tissues) whereas plasma samples were normalized against the VHSV08-assay. Water samples were normalized against both the Sal-assay and the VHSV08-assay. Samples from dead fish were not normalized, but only considered as SAV-positive or SAV-negative. RNA extraction controls and no template controls (NTC) were included in all runs in order to detect possible contamination. In addition, a positive control was included in all runs in order to detect reagent mix errors. If water or plasma samples were found SAV-positive, one parallel sample that had not been spiked with VHSV was also checked for the presence of VHSV that could have given rise to false background for normalization of real-time RT-PCR data (VHSV-negative controls).

Mean Ct-values for the target gene nsP1 were normalized against endogenous (EF1A_A_)- and exogenous reference genes (Sal- and VHSV-assays) by the use of the Microsoft^®^Excel^® ^based computer software Q-Gene [47]. The resulting mean normalized expression (MNE)-values were transformed into N-folds by defining the lowest MNE valued obtained during the experiment for each tissue as 1. The data were then Log2 transformed. In order to evaluate if there were any significant differences in the normalized RNA levels of samples from the SNorm and the SRed groups, Log2 values for each sample from both groups at the various sampling points were imported into the GraphPad Prism 5.00 software; GraphPad Software, Inc., San Diego, CA. Statistical differences in viral RNA levels in plasma, water, gills and hearts between groups were evaluated by a Kruskal-Wallis non-parametric test followed by Dunn's post test. The same test were also used for evaluating differences in weight, length and condition factor between groups, whereas the Fisher's exact test were used to evaluate mortality levels between groups. A p-value of 0.05 or less was considered as significant.

### Histology and histological scoring system

Tissues (heart, somatic muscle and pancreatic tissue from pyloric caeca region and associated with spleen) from five fish from all experimental groups at six time points were fixed in a modified Karnovsky's fixative containing Ringer's solution with 4% sucrose, and kept at 4°C until further processing. Tissues were washed 3 times (15 min each) in a phosphate buffer with Ringer's solution and dehydrated in an ethanol series (70%-96% ethanol). The tissue were then infiltrated with Historesin (7022 31731 Leica Historesin Embedding Kit) (Leica Microsystems) or with Technovit 7100 (Hereaus Külzner) as described by the manufacturer, and left to harden in molds over night at room temperature. Sections 1.5-2 μm thick were cut on a Reichert- Jung 2050 Supercut microtome (Cambridge Instruments) or a Leica RM2255 and then mounted on slides in dH_2_0. Sections were dried and stained with 1% Toluidine blue and studied in a Leitz Dialux 20 or a Leitz Aristoplan light microscope (Leica). Pictures were taken with a digital Olympus camera E-330 or a Nikon DS-US1 camera with NIS-Elements software version 5.03 (Nikon Instruments Inc). Histopathological lesions in pancreas, muscle and hearts were evaluated from five fish from each group at each time point from the SNorm and SRed groups (n = 60), whereas three fish from each group at each time point were evaluated from CNorm and CRed (n = 40). Lesions were scored in accordance to a semi-quantitative lesion score system (Table 1) based upon the one presented by McLoughlin et al (2006) [22]. Briefly, normal histology was given the score 0, focal to mild pancreatic acinar cell degeneration/myocytic degeneration in hearts and muscle (± inflammation) were given the score 1, whereas score 2 and 3 depicted more severe lesions in the tissues (see Table 1 for details). Only lesions with a score of ≥ 2 were considered as PD-specific as focal epicarditis (score 1) could also be seen in hearts from some of the fish in the control groups. Lesions were evaluated as a blind study.

**Table 1:** Semi-quantitative score system for comparing lesion severity between tissues.

Score	Description
**Pancreas lesions**	
0	Normal appearance
1	Focal pancreatic acinar cell degeneration ± inflammation
2	Multifocal degeneration/atrophy of pancreatic acinar tissue, plus some normal tissue left ± inflammation
3	Significant multifocal degeneration/atrophy of pancreatic acinar tissue, no normal tissue left ± inflammation
**Heart lesions**	
0	Normal appearance
1	Focal myocardial degeneration and/or inflammation (< 50 fibres affected)
2	Multifocal myocardial degeneration ± inflammation (50-100 fibres affected)
3	Severe diffuse myocardial degeneration ± inflammation (> 100 fibres affected)
**Muscle lesions**	
0	Normal appearance
1	Focal myocytic degeneration ± inflammation
2	Multifocal myocytic degeneration ± inflammation
3	Severe diffuse myocytic degeneration± inflammation

The system was adapted from McLoughlin et al (2006) [22] with some modifications.

## Results

### Real-time RT-PCR standard curves and efficiencies

The PCR efficiency, regression analysis and standard curve slope s (Ct-value vs. log quantity) of the various assays were calculated from the Ct-values obtained from dilution series of RNA and are given in Table 2. The mean slope for all assays was similar (Table 2) and indicated high PCR efficiency.

**Table 2:** Standard curve evaluation of the various assays.

Assay	Slope	**R**^**2**^	E
nsP1	-3.6652	0.9975	0.8743
EF1A_A_	-3.6711	0.9991	0.8724
SAL	-3.7425	0.9849	0.8501
VHSV08	-3.3961	0.9699	0.9839

The mean slope of the standard curve, regression (R^2^) and efficiency (E = -1+10(-1/slope)) were calculated.

### Bacteria isolations

Several bacteria were isolated on marine agar and blood agar (2% NaCl) from head kidney of salmon during this experiment. By BLAST search tool the bacteria were identified to genus and it was established that they all belonged to marine genera; *Idiomarina sp*., *Cobetia marina, Janibacter sp., Bacillus sp., Tenacibaculum/Flavobacterium, Vibrio sp., Vibrio splendidus*, and *Pseudoalteromonas sp*.

### Oxygen levels

Two days after infection, the oxygen levels in one tank with uninfected fish (CRed) and one tank with SAV-infected fish (SRed), were gradually (slowly within a 24 h period) lowered from 85-90% saturation (9.2-9.7 mg O_2 _per liter, approx. 300-400 lh^-1^tank^-1^) to 60-65% saturation (6.5-7.0 mg O_2 _per liter, approx. 100-200 lh^-1^tank^-1^) by reduction of the flow-through rate. The average oxygen level during the experiment in the various experimental tanks was; CNorm; mean 86.29 (min 80, max 94), CRed; mean 64.79, (min 52, max 71), SNorm; mean 84.32 (min 69, max 93), SRed; mean 65.57 (min 57, max 75), respectively.

### Mortality

**Figure 1:**
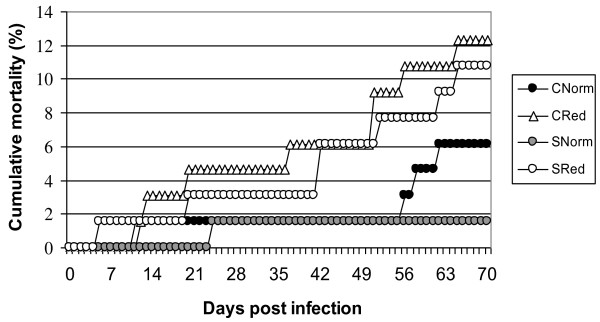
**Cumulative mortality and morbidity during the experiment**. The number of fish in each tank was 65. The highest percentage of mortalities/morbidities could be seen in the CRed (8 out of 65; 12.3% morbidity/mortality) and in the SRed group (7 out of 65; 10.8% morbidity/mortality). CNorm = Uninfected controls, normoxia. CRed = Uninfected controls, reduced oxygen conditions. SNorm = SAV-infected, normoxia. SRed = SAV-infected, reduced oxygen conditions.

Mortality data were collected on a daily basis, and cumulative mortality ranged from 6.1% (CNorm) to 12.3% (CRed) in the uninfected controls, and from 1.5% (SNorm) to 10.8% (SRed) in the SAV-infected groups (Figure 1). The differences in mortalities between groups were tested with a Fisher's exact test, which found no statistical significant differences between groups. All dead fish (gill tissue) were analyzed with real-time RT-PCR; of these no fish in the control groups were SAV-positive, whereas in the SAV-infected groups there was one out of one (SNorm) and five out of seven dead fish (SRed) which were SAV-positive (raw Ct-values of 29-37).

### Clinical signs and gross pathological changes

Variable degrees of fin erosions (sometimes with bleedings), especially of the dorsal and pectoral fins, could be seen in all experimental groups throughout the study. Some individuals also had petecchial bleedings/erythemia on abdomen and at pectoral fin bases, and four dead fish had severe erosions behind pectoral fins (CRed and SRed groups).

### Weight and length development (condition factor)

During the 70 days the experiment lasted, it was not possible to see a significant increase in mean body length or weight for the fish groups, nor a considerable difference in mean weight, length or Fulton's condition factor (K) between the various groups (see additional files 1, 2 and 3).

### SAV in blood

**Figure 2:**
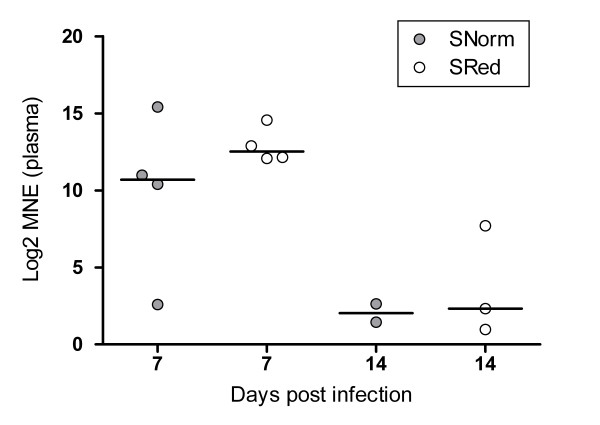
**Levels of SAV-specific viral RNA in plasma**. Viral RNA (nsp1-assay) was normalized against the exogenous spike VHSV. Mean normalized expression (MNE) values were Log2 transformed. Plasma samples were only positive at 7 and 14 dpi. At 7 dpi; 4/4 were SAV-positive in both SAV-groups, whereas 2/5 and 3/5 plasma samples were SAV-positive at 14 dpi in the SNorm and SRed, respectively. Significant differences between the SAV- groups at each time point were tested with a Kruskal-Wallis non-parametric test followed by a Dunn's post test. SNorm = SAV-infected, normoxia. SRed = SAV-infected, reduced oxygen conditions. Median values are shown as horizontal lines.

No viral RNA from SAV was detected in plasma samples from the control groups at any time points. In both SAV-infected groups, SAV was detected in plasma samples by real-time RT-PCR in fish sampled at 7 and 14 dpi only. Viral RNA levels were normalized against the exogenous control VHSV (VHSV08-assay) (Figure 2). This normalization strategy demonstrated that the highest levels of SAV nucleic acids occurred at 7 dpi, in both SAV-groups. No statistical differences between SAV groups at each time point could be seen with regards to viral RNA levels. No VHSV-controls (plasma samples without VHSV-spike) analyzed from plasma were VHSV-positive.

### SAV levels in gills and hearts

**Figure 3:**
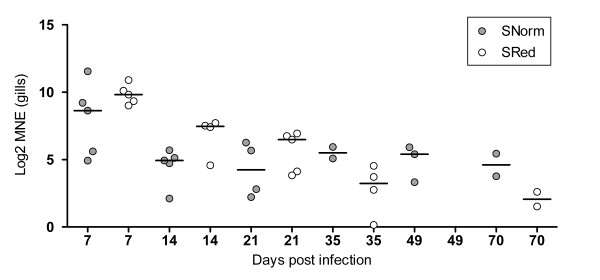
**Levels of SAV-specific viral RNA in gills**. Viral RNA (nsp1-assay) was normalized against EF1A_A_. Mean normalized expression (MNE) values were Log2 transformed. Five fish were sampled from each group at each time point (7, 14, 21, 35, 49 and 70 dpi). N positive at 21, 35, 49 and 70 in the SNorm group were 4, 2, 3 and 2, whereas n positive in the SRed-group at 14, 49 and 70 dpi were 4, 0 and 2, respectively. Significant differences between the SAV- groups at each time point were tested with a Kruskal-Wallis non-parametric test followed by a Dunn's post test. SNorm = SAV-infected, normoxia. SRed = SAV-infected, reduced oxygen conditions. Median values are shown as horizontal lines.

**Figure 4:**
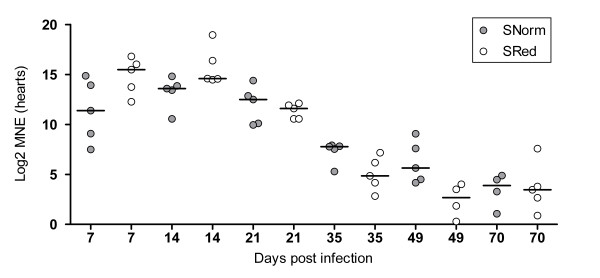
**Levels of SAV-specific viral RNA in hearts**. Viral RNA (nsp1-assay) was normalized against EF1A_A_. Mean normalized expression (MNE) values were Log2 transformed. Five fish were sampled from each group at each time point (7, 14, 21, 35, 49 and 70 dpi). In the SRed group at 49 dpi only 4 fish were positive. SNorm = SAV-infected, normoxia. SRed = SAV-infected, reduced oxygen conditions. Significant differences between the SAV- groups at each time point were tested with a Kruskal-Wallis non-parametric test followed by a Dunn's post test. Median values are shown as horizontal lines.

No SAV viral RNA could be detected in gills from the uninfected groups reared at normal or reduced oxygen levels. However, in hearts from the same individuals, very low amounts of SAV viral RNA could be detected in 7 out of 60 fish at 7-21 dpi. The raw Ct-values were in the range of 32-36. In both SAV-infected groups, viral RNA from SAV was detected in gills (Figure 3) and hearts (Figure 4) at all sampling points. The RNA levels in the gills and hearts peaked at 7 and 14 dpi, respectively (Figure 3 and 4). In general, the viral RNA levels seemed to be higher in hearts compared to the gills. The mean levels of SAV viral RNA when normalized against the reference gene EF1A_A _were not significantly different between the SAV-infected groups in gills or hearts during the experiment.

### Water samples

In order to obtain more detailed information on the onset and duration of the virus shedding period, water from the CNorm and the SNorm groups were monitored more closely than CRed and SRed. Viral RNA was detected in water sampled from the SNorm group between 4-10 days post infection, and in SRed virus were detected at 6 and 13 dpi (Table 3). After this period, SAV specific viral RNA could not be detected in water from any of the SAV-infected groups. SAV were not detected in water samples from the tanks with the uninfected controls. When normalizing the relative amount of viral RNA in water against the spiked filtration control *H. salinarum *and the RNA-extraction control VHSV, it was evident that the highest amounts of viral RNA in water in both SAV-infected groups could be seen at 6 days post infection, declining at 10-13 dpi (Table 3). Only water samples from the SNorm group were normalized against VHSV. No statistical difference in viral RNA levels between groups could be seen.

**Table 3:** Levels of SAV-specific viral RNA (nsp1-assay) in water.

	Log2 MNE nsP1 vs. Sal (VHSV) in water					
	
Groups	2	4	6	8	10	13
**SNorm**	0 (0)	4 (0)	8 (5)	5 (1)	4 (0)	0 (0)
**Sred**	ND	ND	9 (ND)	ND	ND	2 (ND)

nsP1 were normalized against the exogenous spikes *Halobacterium salinarum *and VHSV (parenthesis). Mean normalized expression (MNE) values were Log2 transformed. Viral RNA specific for SAV could only be found in water 4-13 dpi. After this period, no SAV could be detected in water. Only water samples from the SNorm group were normalized against VHSV. SNorm = SAV-infected, normal oxygen conditions. SRed = SAV-infected, reduced oxygen conditions. ND = no data.

### Histopathology

Lesions in pancreatic- and heart tissue were only observed in SAV3-challenged fish. The exception was small foci with epicarditis in hearts, which could be found in 11 out of 43 fish (25.6%) of the examined individuals from the control groups. No muscle lesions were found in any of the experimental groups throughout the study. Two moribund fish were sampled from CNorm at 56 and 62 dpi, and one fish from SRed at 42 dpi. Pancreas, heart and muscle tissue were processed for histology and examined, with no lesions recorded in these organs.

#### Pancreas

**Figure 5:**
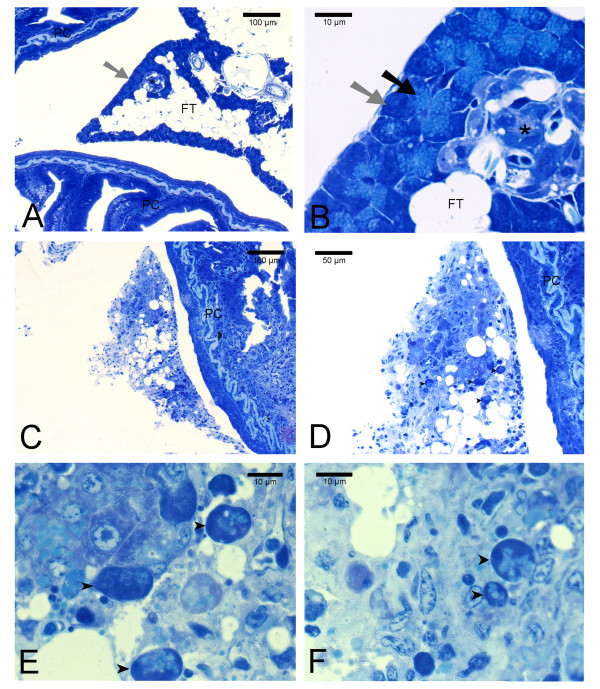
**Pancreas *Salmo salar *L**. Resin sections (1.5 μm) were stained with Toluidine blue. A) and B); normal pancreatic tissue with fat tissue (FT) between pyloric caeca (PC). Note endocrine pancreas; islets of Langerhans (_*_). Zymogene granula (black arrow) can be seen inside the exocrine pancreas acini (grey arrows). C)- F); pancreatic tissue from SAV-infected fish (7 dpi) showing rounding and vacuolation (arrowheads) of exocrine acini/cell degeneration. Note that zymogene granula can still be seen in some degenerated acini.

Vacuolation and rounding of the acinar cells could be seen in 5 out of 8 individuals at 7 dpi. (Figure 5). Degeneration and fibrosis of the exocrine part of the pancreatic tissue was observed in 8 fish at 7-21 dpi. After this period no pancreatic lesions were evident.

#### Hearts

**Figure 6:**
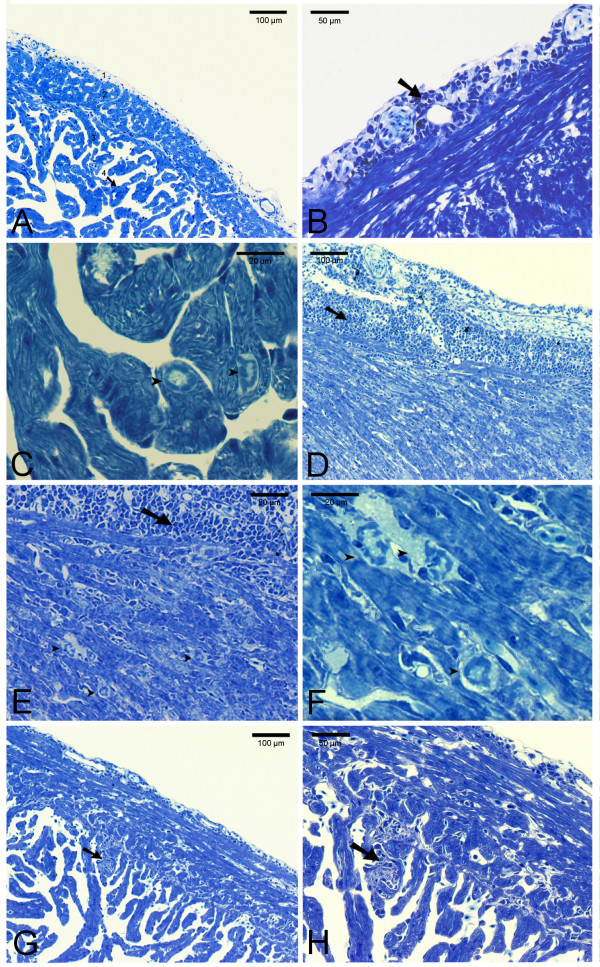
**Hearts *Salmo salar *L**. Resin sections (1.5 μm) were stained with Toluidine blue. A); normal heart tissue, showing the various layers of the ventricle; epicardium (1), *stratum compactum *(2) and *stratum spongiosum *(3) together with endocardium (4). B) -H); ventricle from SAV-infected fish. Focal epicarditis (B; black arrow) and focal cardiomyocytic degeneration (C; arrowheads) could be observed at 14 dpi. D)-F); severe epicarditis and myocarditis (black arrows), together with multifocal degeneration of cardiomyocytes (arrowheads) was seen at 21 dpi. G) and H); hypercellularity and inflammation (black arrows) in the contact layer between compact and spongy myocardium was seen at 35 dpi.

Focal epicarditis of the ventricle was found in some individuals at 7-14 dpi in both SAV-infected groups (Figure 6). At 14 dpi, focal to multifocal cardiomyocytic degeneration was identified in compact and spongy layers of the ventricle in a few individuals (score 2). In the SNorm- and the SRed groups, severe epicarditis could be seen in hearts at 21 dpi, together with severe multifocal necrosis and inflammation of the compact and spongy myocardium (score 3). At 35 dpi, a moderate to extensive multifocal epicarditis was present. Infiltration of inflammation cells/increased cellularity could be seen, especially in the junction of compact and spongy myocardium at 35-49 dpi. In this period, sporadic focal degeneration and inflammation of myocard was found. After 35 dpi, only small foci with epicarditis could be seen together with foci of increased cellularity/inflammation in the junction of compact and spongy layers of the ventricle.

Results from histopathological examination of pancreas, heart and skeletal muscle of all experimental groups was evaluated in a semi-quantitative approach (Table 1) based upon the scoring system described in McLoughlin et al (2006) [22] with some modifications. Findings are summarized in Table 4. Severe lesions of score 3 in heart (severe inflammation together with multifocal to diffuse cardiomyocytic degeneration in ventricle) could be seen at 21-35 dpi in both SAV-infected groups during the experiment. No difference in histo-score was found between SNorm and SRed at any sample point.

**Table 4:** Summary of score lesions (histopathological findings) from heart tissue.

	Days post infection					
Groups	7	14	21	35	49	70

SNorm	0	0	3	2	0	0
	0	2	2	2	0	0
	0	2	3	3	2	0
	0	2	0	2	0	2
	0	2	ND	2	0	0

	0%	80% (1.6 ± 0.9)	75% (2 ± 1.4)	100% (2.2 ± 0.5)	20% (0.4 ± 0.9)	20% (0.4 ± 0.9)

SRed	0	0	3	2	0	0
	ND	2	0	2	0	0
	0	2	3	2	0	0
	0	2	3	2	0	0
	0	0	3	3	0	0

	0%	60% (1.2 ± 1.1)	80% (2.4 ± 1.3)	100% (2.2 ± 0.5)	0%	0%

For a description of score criteria, see Table 1. Hearts from five fish were sampled from each group at each time point. No lesions could be seen in control fish, except for small foci with epicarditis in 11 out of 43 control fish (25.6%). The percentage of fish with PD-specific lesions (≥2) at each time point is given, and also the mean lesion score with standard deviation given in parenthesis. SNorm = SAV-infected, normal oxygen conditions. SRed = SAV-infected, reduced oxygen conditions. ND = no data.

## Discussion

A large discrepancy exists between the high mortality levels often reported with pancreas disease (PD) in field versus experimental infections with SAV. It is possible that certain key environmental factors, such as oxygen levels, might play an important role concerning the severity or the progress of PD. In general, hypoxia can act as a stressor to fish [35] which may result in impaired immune functions mediated through the hypothalamo-pituitary-interrenal axis and lead to decreased resistance to infections [48,49]. In our study, mortality could not be linked directly to the oxygen levels since similar mortality levels could be seen in all experimental groups. Furthermore, the development or progress of PD was not affected by oxygen levels as lesions of comparable severity were seen during the same time period in both SAV-groups. It is possible that by subjecting fish to constant sub-lethal oxygen levels as performed in this trial, the fish probably experienced a lower stress level and were able to acclimatize to the hypoxic conditions. If the salmon had been repeatedly exposed to fluctuating oxygen levels and not constant suboptimal levels, it is possible that this would have affected the disease progress or led to higher mortality levels (i.e added stress). Exposure of fish to hypoxic conditions for a longer time prior to SAV-infection than was used in this study could have rendered the fish more susceptible to SAV-infection. Moreover, it can not be excluded that the virus infection route could have an impact on mortality levels, as this has been seen for another salmon virus, IPNV, a feature which was attributed to the immune system being activated in different ways [50].

The SAV-infection led to severe lesions in hearts during the course of infection, characterized by epicarditis and multifocal degeneration of cardiomyocytes in ventricle of the heart. Ferguson et al [51] concluded that the most severe lesions associated with PD were myocardial degeneration. In our study, in addition to severe lesions in hearts, lesions in exocrine pancreas could also be seen at 7-21 dpi, whereas no lesions could be seen in skeletal muscle during the experiment. It is possible that the absence of muscle lesions in many experimental studies could explain the lower mortality levels reported from experimental SAV-infections, as muscle lesions have been suggested as a contributing factor to PD-mortality in field [52]. Furthermore, muscle lesions in the oesophagus have been reported from PD-cases [51,52], a feature which probably has the potential to interfere with food intake. Nevertheless, the presence of muscle lesions is probably not the single reason for the discrepancy in mortality levels, since experimental SAV-studies have been described where muscle lesions were induced but with no mortality observed [4,28].

Salmon has been shown to produce protective neutralizing antibodies shortly after i.p infection [53], readily diminishing viruses from the system. SAV-specific viral RNA was shown to be present in tissues (gills and hearts) at lasting low levels after the acute phase and throughout the experimental period of 70 days. Such long-lasting presence of SAV-specific viral nucleic acids in tissues have previously been described by Christie et al [28] (140 days) and by Andersen et al [45] (190 days) during experimental infections, and also in longitudinal field studies [16,54]. The nature of these SAV-specific RNAs has not been determined [28,45]. A few fish from the control groups were shown to be SAV-positive (heart tissue) in this study, which might be due to carrier status as presence of SAV in the fresh water phase has been shown [55,56].

This study extends the current knowledge of SAV-pathogenesis in Atlantic salmon since it is the first to demonstrate virus shedding during infection. The present study also shows that virus shedding coincides with viraemia. Atlantic salmon *Salmo salar *L. smolts that were intraperitoneally (i.p.) infected with a salmonid alphavirus (SAV3) isolate (SAVH30/04) shed detectable levels of virus into the surrounding water in the period 4-13 days post infection. This was assessed by a VIRADEL (virus-adsorption-elution) method using electropositive 1MDS virus filters (see [40-43]). The VIRADEL method was optimized for downstream real-time RT-PCR and this modified method has proven to be a very useful tool which can supplement experimental infection trials with information related to viral shedding. Also, this method has a potential for simultaneous detection and monitoring of levels of other pathogens, as such abilities have been demonstrated for electropositive 1MDS filters [43]. Detection of infectious SAV-particles in water samples can also be monitored by inoculation onto susceptible cell cultures after viruses have been eluted from filters with cell medium containing sera. We further demonstrate that the highest levels of SAV-specific viral RNA shed to the water could be seen at 6 days post infection in both SAV-infected groups. This coincided with the period where the highest SAV viral RNA levels could be seen in plasma (7 dpi) and gills in both SAV-groups. Both infectious SAV-particles/viral RNA have been shown to only be present in blood for a relative short period after infection [54]. The presence of viral RNA in plasma as soon as 7 dpi is suggestive of a rapid onset of viraemia after i.p. injection of virus, as reported for other experimental i.p. infections with SAV [22,27,28,57]. In tissues, the highest viral RNA levels specific for SAV could be seen at 7 dpi in gills and at 14 dpi in hearts, respectively. In general, the viral RNA levels seemed higher in hearts than in gills. However, no significant differences were found between the SAV-infected groups regarding viral RNA levels in tissues or water, suggesting that oxygen levels did not have a considerable effect on the infection. In our study, detectable levels of virus shedding preceded appearance of severe lesions in heart which could be seen from 21 -35 days after i.p. infection. Viral shedding from a SAV-infected fish population in a farm, however, will be a more complex situation, as the time period where viral shedding can be seen will be more prolonged and not a synchronous event as seen in experimental tank systems. In addition, information is lacking regarding the route of virus entry/exit and the virus dose necessary to elicit an infection. Our findings that SAV shedding coincides with blood viraemia further supports the proposal of Graham and coworkers [54] that in order to detect or monitor an active SAV-infection in a given salmon population, blood serum or plasma should be monitored in addition to the previously recommended tissues for real-time RT-PCR diagnostics; pseudobranch/gills and hearts [45,54].

## Conclusions

In the present study, experimentally induced hypoxia failed to explain the discrepancy between the severities reported from field outbreaks of PD-disease and experimental infections as the severity or the progress of the disease was not affected. We also demonstrate for the first time by the use of a modified VIRADEL method that detectable levels of SAV are shed into water during viraemia.

## Competing interests

The authors declare that they have no competing interests.

## Authors' contributions

AN and LA designed the experiment and conducted the fish infection. LA designed the modified water filtration method and performed all laboratory work, except the design of the Sal-assay, which was made by KH. LA and AN evaluated the histology sections. LA analyzed the results and wrote the manuscript. AN and KH critically revised the manuscript. AN has contributed with discussions during planning. All authors read and approved the final manuscript.

## Supplementary Material

Additional file 1 **K-factor**. The additional files K-factor.jpg, length.jpg and weight.jpg describe mean development of condition factor (K), length in cm and weight in grams for all groups during the experiment. Click here for file

Additional file 2 **Length**. The additional files K-factor.jpg, length.jpg and weight.jpg describe mean development of condition factor (K), length in cm and weight in grams for all groups during the experiment. Click here for file

Additional file 3 **Weight**. The additional files K-factor.jpg, length.jpg and weight.jpg describe mean development of condition factor (K), length in cm and weight in grams for all groups during the experiment. Click here for file
