# Supplementary figures and images for: No influence of oxygen levels on pathogenesis and virus shedding in Salmonid alphavirus (SAV)-challenged Atlantic salmon (Salmo salar L.)
Source: Virol J. 2010 Aug 21;7:198. doi: 10.1186/1743-422X-7-198 (PMC2936311; doi:10.1186/1743-422X-7-198)

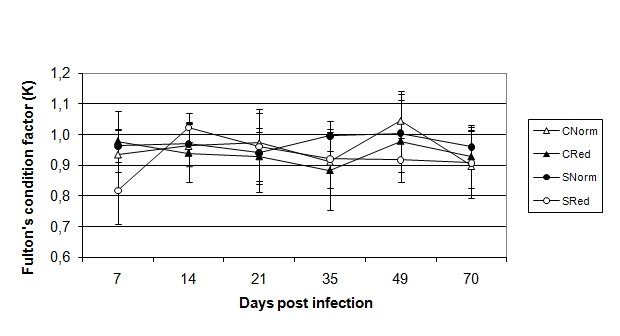

Supplement: Additional file 1 — K-factor. The additional files K-factor.jpg, length.jpg and weight.jpg describe mean development of condition factor (K), length in cm and weight in grams for all groups during the experiment. [file 1743-422X-7-198-S1.JPEG]

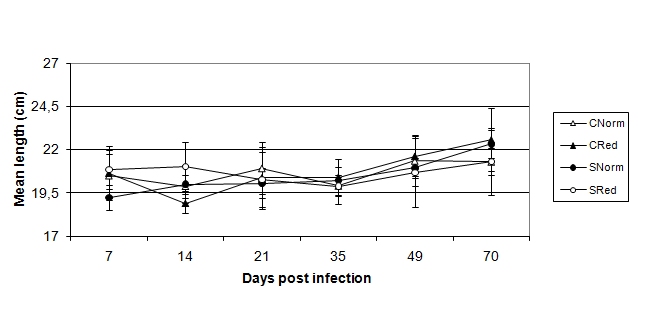

Supplement: Additional file 2 — Length. The additional files K-factor.jpg, length.jpg and weight.jpg describe mean development of condition factor (K), length in cm and weight in grams for all groups during the experiment. [file 1743-422X-7-198-S2.JPEG]

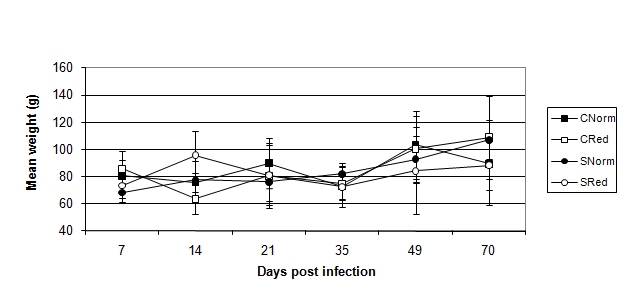

Supplement: Additional file 3 — Weight. The additional files K-factor.jpg, length.jpg and weight.jpg describe mean development of condition factor (K), length in cm and weight in grams for all groups during the experiment. [file 1743-422X-7-198-S3.JPEG]
